# Genomic characterization of tobacco/nut chewing HPV-negative early stage tongue tumors identify *MMP10* as a candidate to predict metastases

**DOI:** 10.1016/j.oraloncology.2017.08.003

**Published:** 2017-10

**Authors:** Pawan Upadhyay, Nilesh Gardi, Sanket Desai, Pratik Chandrani, Asim Joshi, Bhaskar Dharavath, Priyanca Arora, Munita Bal, Sudhir Nair, Amit Dutt

**Affiliations:** a Integrated Genomics Laboratory, ACTREC, Tata Memorial Centre, Navi Mumbai 410210, India; b Division of Head and Neck Oncology, Department of Surgical Oncology, Tata Memorial Hospital, Tata Memorial Centre, Mumbai 400012, India; c Homi Bhabha National Institute, Training School Complex, Anushakti Nagar, Mumbai 400094, India; d Department of Pathology, Tata Memorial Hospital, Tata Memorial Centre, Mumbai 400012, India

**Keywords:** HPV-negative early stage tongue cancer, Tobacco/nut chewers, Whole exome and transcriptome sequencing, Nodal metastases, Matrix metalloproteinases

## Abstract

**Objectives:**

Nodal metastases status among early stage tongue squamous cell cancer patients plays a decisive role in the choice of treatment, wherein about 70% patients can be spared from surgery with an accurate prediction of negative pathological lymph node status. This underscores an unmet need for prognostic biomarkers to stratify the patients who are likely to develop metastases.

**Materials and methods:**

We performed high throughput sequencing of fifty four samples derived from HPV negative early stage tongue cancer patients habitual of chewing betel nuts, areca nuts, lime or tobacco using whole exome (n = 47) and transcriptome (n = 17) sequencing that were analyzed using in-house computational tools. Additionally, gene expression meta-analyses were carried out for 253 tongue cancer samples. The candidate genes were validated using qPCR and immuno-histochemical analysis in an extended set of 50 early primary tongue cancer samples.

**Results and conclusion:**

Somatic analysis revealed a classical tobacco mutational signature C:G > A:T transversion in 53% patients that were mutated in *TP53*, *NOTCH1*, *CDKN2A*, *HRAS*, *USP6*, *PIK3CA*, *CASP8*, *FAT1*, *APC*, and *JAK1*. Similarly, significant gains at genomic locus 11q13.3 (*CCND1*, *FGF19*, *ORAOV1*, *FADD*), 5p15.33 (*SHANK2*, *MMP16*, *TERT*), and 8q24.3 (*BOP1*); and, losses at 5q22.2 (*APC*), 6q25.3 (*GTF2H2*) and 5q13.2 (*SMN1*) were observed in these samples. Furthermore, an integrated gene-expression analysis of 253 tongue tumors suggested an upregulation of metastases-related pathways and over-expression of MMP10 in 48% tumors that may be crucial to predict nodal metastases in early tongue cancer patients. In overall, we present the first descriptive portrait of somatic alterations underlying the genome of tobacco/nut chewing HPV-negative early tongue cancer, and identify MMP10 as a potential prognostic biomarker to stratify those likely to develop metastases.

## Highlights

• Portrait of somatic alterations in HPV-negative early stage tongue tumors with tobacco signature.
• Upregulation of genes related to EMT pathway identified by transcriptome analysis.
• *MMP10* could be a candidate prognostic biomarker to stratify patients who develop metastases.

## Introduction

Tongue cancer is the most predominant form of oral cancer in developed countries with a varying incidence in developing countries [1]. The major etiological factors associated with tongue cancer include tobacco related products, alcohol and human papilloma virus (HPV) infections [2]. These factors lend to variability across populations, particularly in the Indian subcontinent wherein chewing betel-quid comprising betel leaf (Piper betel), areca nut (*Areca catechu*) and slaked lime (predominantly calcium hydroxide) is a part of the tradition [3]. While tobacco usage shows a 5–25-fold increased risk of cancer [4], HPV infection defines clinical and molecularly distinct subgroups of head and neck squamous cell carcinoma (HNSCC) patients [5]. Such as, HPV-negative tumors are driven by amplification at 11q13, *EGFR* and *FGFR* loci; focal deletions at *NSD1*, *FAT1*, *NOTCH1*, *SMAD4* and *CDKN2A* loci; and, point mutations in *TP53*, *CDKN2A*, *FAT1*, *PIK3CA*, *NOTCH1*, *KMT2D*, and *NSD1* [6], [7]. On the other hand, HPV-positive tumors harbor *TRAF3*, *ATM* deletion, *E2F1* amplification, *FGFR2/3* and *KRAS* mutations.

Another unique feature of tongue squamous cell carcinoma (TSCC) compared to other subsites of oral cancer is the occurrence of nodal metastases, observed in 27–40% of early stage (pT1 or pT2) patients. Neck dissection among them adds to morbidity and poor survival due to disease recurrence [8], [9], [10], [11]. Although poor prognostic indicators for TSCC such as occult node positivity, tumor depth, lymphovascular invasion and perineural invasion are well defined, there’s an unmet need for reliable and robust prognostic biomarkers to stratify the patients who are likely to have an adverse clinical outcome [11], [12]. Interestingly, most of the genomic analysis studies involving HPV negative TSCC have been restricted to advanced stage samples (pT3–pT4), while genomic alterations underlying HPV negative early tongue tumor genome remains largely unexplored. In the present study, we present a portrait of somatic alterations in HPV negative early tongue cancer (pT1–pT2) using integrative genomic approach to identify marker to stratify those likely to develop metastases.

## Material and methods

### Sample selection and patients details

The sample set and study protocol were approved by (ACTREC-TMC) institutional Internal Review Board. All the tissue samples used under study have been taken after obtaining informed consent from patients. Primary tongue tumors were staged as T1 (measuring ≤2 cm) or T2 (measuring >2 cm but <4 cm) as per AJCC (American Joint Committee on Cancer)/UICC (Union for International Cancer Control) TNM classification (7th edition) system and primary tumors with early stage (T1 and T2) were included in this study. Samples were duly verified by two independent reviewers for histological examinations such as normal sample verification, percent tumor nuclei and lymph node metastasis. The tumor sample with concordant histopathological diagnosis by both reviewers was included in the study. Tumor with at least >50% tumor nuclei was used for data analysis. Clinical, histological and etiological features were collected along with follow-up data for recurrence (Supplementary Table 1). None of the samples showed the presence of HPV using HPVDetector and PCR-based validation using the MY09/11 method as described previously [13], [14].

### Exome capture and NGS DNA sequencing

Exome capture and sequencing were performed as described previously [14]. Briefly, TruSeq Exome Enrichment kit (Illumina) and NimbleGen SeqCap EZ Exome Library v3.0 were used to capture ∼62 Mb region of human genome comprising of ∼201,121 exons representing ∼20,974 gene sequences, including 5′UTR, 3′UTR, microRNAs and other non-coding RNA.

### Somatic variant analysis, functional annotations and prioritization

The variant analysis was performed as described previously [14] and detailed in supplementary material and methods. MutSigCV v2.0 [15] and IntOgen [16] were used for identification of the significantly mutated gene and p value ≤ 0.05 was considered as the threshold for significance, as described earlier [17], [18]. Since our dataset was inherently not suitable for above tools due to a limited number of samples (n = 25), we have also performed extensive functional prediction tool based analysis for non-synonymous variants using nine different tools (detailed in supplementary material and methods). The total number of identified somatic substitutions in exome sequencing were extracted from the MutSigCV output and were processed to calculate the number and frequency distribution of various transitions and transversions.

### Somatic copy number analysis from exome sequencing data and qPCR validation

DNA copy number analysis of exome sequencing data was performed as described previously [14] and detailed in supplementary material and methods. Genes with Segments-of-Gain-Or-Loss (SGOL) score ≥4 were defined as amplified genes and ≤−2 as deleted genes. The validation of somatic copy number changes was performed as described previously [14]. Details of the primers used for copy number study are provided in Supplementary Table S15.

### Transcriptome sequencing and data analysis to identify expressed genes

Transcriptome libraries for sequencing were constructed according to the TruSeq RNA library protocol (Illumina) outlined in TruSeq RNA Sample Prep (Illumina) performed as described previously [14] and detailed in supplementary material and methods. Transcriptome data analysis was performed using previously published a protocol for transcriptome sequencing data analysis [19]. First, to identify the bona fide expressed transcripts, we filtered all the transcripts which were lowly expressed (≤0.1 log 10 (RSEM + 1)) for each sample; second, transcript expressed in 10% of samples was considered as a candidate expressed gene in tongue tumor tissue. A list of 16,525 transcripts identified to be expressed in TSCC tumors were used to filter mutation and DNA copy number changes in this study.

### Gene expression dataset meta-analysis and RT-qPCR validation

TSCC gene expression profiling studies were identified by searching Gene Expression Omnibus (GEO) database [20] using keyword ‘tongue cancer’, ‘tongue tumor’. The TCGA-HNSCC dataset were downloaded from Cancer Genome Browser [21] and tongue sub-site data was extracted for the analysis. The criteria for selection of data set included fresh frozen tumor samples with corresponding normal sample and studies with >10 patient samples in the cohort. Studies involving cell lines, <10 samples, and non-human tissue samples were excluded. Raw data from 4 GEO microarray based data (GSE34105, GSE13601, GSE9844, and GSE31056) was analyzed using BRB array tool [22]. Briefly, non-variable genes were excluded from the analysis based on the log expression variation filter (variance of a gene across the arrays) followed by a class comparison of samples based on normal verses tumor comparison. Genes were considered as differentially regulated if a gene followed 1.5 <fold change <−1.5 filter along with p-value <0.05. Gene set enrichment analysis (GSEA) was performed selecting KEGG gene set in MSigDB [23] to identify underlying biological processes and pathways. The validation of gene expression was performed using quantitative reverse transcriptase PCR analysis as described previously [24] [14]. The primer sequences for genes are provided in Supplementary Table S16.

### Immunohistochemical analysis

Immunohistochemical staining, was performed with the help of pathologist as described previously [14] and is detailed in supplementary material and methods.

## Statistical analysis

The clinicopathologic association analysis was performed using IBM SPSS statistics software version 2. Test for overlap significance for a number of genes overlap for copy number changes different studies and databases were carried out using previously described method (http://nemates.org/MA/progs/representation.stats.html). The mutual exclusivity and co-occurrence analysis were performed using Gitools [25]. The significant differences between selected two groups were estimated using Unpaired Student *t*-test and P-value <0.05 was considered as a threshold for statistical significance.

## Results

### Patient details

Fifty-seven patient-matched normal early tongue cancer patient tumor were analyzed for somatic mutations, copy number changes, and differential expression by whole exome and transcriptome sequencing approach (Supplementary Fig. S1a, b). The clinicopathological details for fifty-seven in the cohort are detailed in Supplementary Table S1. In brief, our cohort comprised of 72% male; 61% tobacco users; 80% chewers of either betel, tobacco, areca, or lime; and, 28% smokers, with a median age of 45 years (range 25–72 years). 56% of all patients with pT1 and pT2 staged tongue tumors were lymph node positive (n = 32) at the time of registration. Primary treatment modality for all the patients was surgery followed by radiation and chemotherapy alone or in combination with chemo-radiation therapy. None of the patients were positive for HPV infection as reported previously [13], [14]. Forty patient follow-up data was available and median survival duration for the cohort was 29 months (range 2–42 months). During this time period, there were 9 recurrences, 6 metastasis and 8 fatal events.

### Somatic variants in HPV negative early tongue squamous cell carcinoma

We performed whole exome sequencing of forty seven samples including early TSCC tumor (n = 24) and matched normal (n = 23) samples. We captured ∼62 Mb of coding genome at a median coverage of 97x for tumor samples and 103x coverage for normal samples. Somatic variants were called using Mutect [26] and GATK algorithm [27]. We identified a total of 2969 somatic mutations across 19 TSCC patients (5 patients were excluded from further analysis due to low coverage and/or poor correlation with their matched normal), which included 1693 missense, 60 nonsense, 972 silent, 124 splice site as well as 120 indels. The aggregated non-silent mutation rate across the dataset was 4.12 per Mb, consistent with the literature [28], [29]. The sequencing statistics and somatic mutational features are provided in Supplementary Tables S2–S3 and Supplementary Fig. 2a–e.

**Fig. 1:**
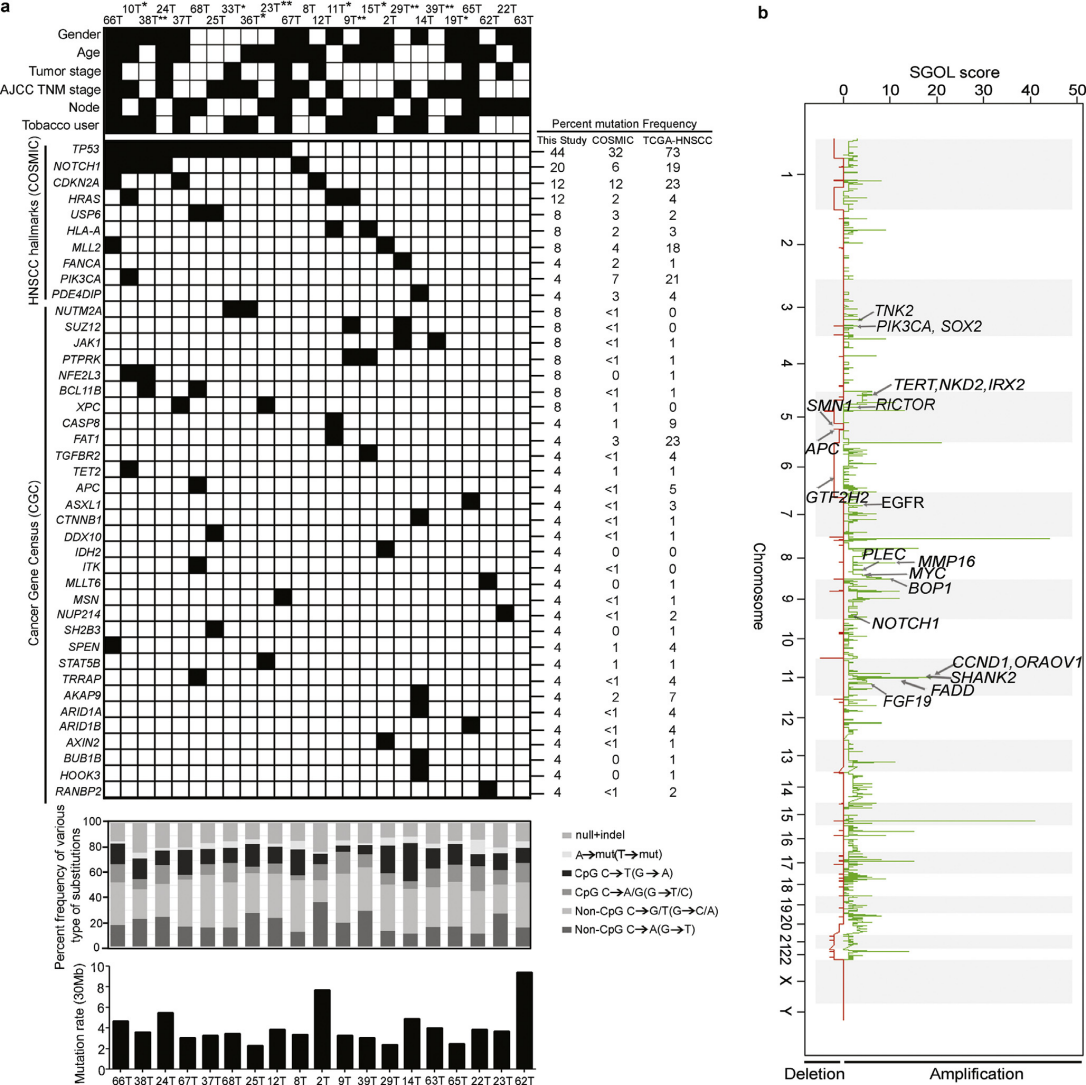
Identification of somatic mutations and DNA copy number changes in HPV-negative early stage TSCC tumors. (a) Mutational features of 25 early tongue squamous carcinoma samples: 19 of 24 whole exome (5 samples excluded due to low quality reads) and 6 of 11 whole transcriptome sequencing (excluding 5 common samples). Samples ID’s with asterisk (**) denotes samples with exome and transcriptome sequencing; (*) samples with transcriptome sequencing alone. Different clinicopathological factors such as; gender, age, tumor stage, AJCC TNM stage and lymph node metastasis status and etiological factors such as tobacco users are shown for each patient. The black solid boxes denotes gender: male, age: >45 years, tumor stage: pT1, AJCC-TNM stage: Stage I–II, nodal status: positive and tobacco habit. The white boxes denote gender: female, age: <45 years, tumor stage: pT2, AJCC-TNM stage: Stage III–IV, nodal status: negative and without tobacco habit. Grey filled boxes denotes no information available. The ten HNSCC hallmark genes and cancer gene census (COSMIC) found to be mutated in data, is arranged in decreasing order of percent frequency. Black filled box denotes presence of a somatic mutation in the patient. Mutation frequencies for the hallmark and cancer census genes observed in this study (n = 25), COSMIC-HNSCC (n ≥ 500) and TCGA-HNSCC (n = 279) samples. The substitution frequencies spectrum for each patient for whole exome sequencing data is shown. Percent frequency of various types of SNVs and indels are shown. Different types of substitutions shown by different shades. Somatic non-silent mutation rate/30 Mb derived from whole exome sequencing data for each tumor is shown. (b) Somatic DNA copy number changes identified using Exome sequencing data. Somatic DNA copy number gains and losses were generated using Segments-of-Gain-Or-Loss (SGOL) scores across 23 TSCC patients. SGOL score is plotted (horizontal axis) for DNA copy number gains (green) and losses (red) are plotted as a function of distance along with human genome (vertical axis). Representative amplified and deleted regions are annotated for HNSCC-associated genes and denoted by an arrow. (For interpretation of the references to color in this figure legend, the reader is referred to the web version of this article.)

**Fig. 2:**
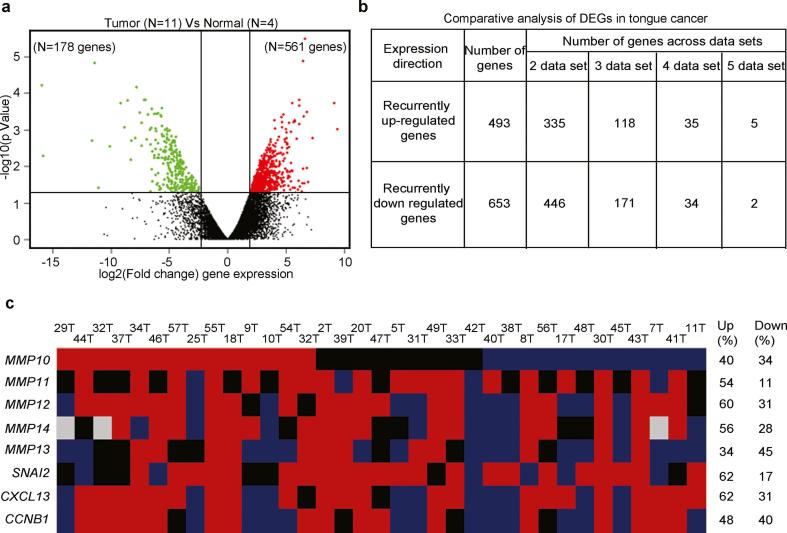
Differential expression profile of tongue squamous cell carcinoma using mRNA sequencing and meta-analysis identifies *MMP10* up regulation. Differential expression analysis to identify the distinct gene expression profile of tongue tumors. (a) Volcano plot representation of differentially expressed in between early tongue tumors and adjacent normal tongue tissues. The red and green dots denote the up-regulated and down-regulated differentially expressed genes with P value <0.05 and fold changes ≥2 or ≤−2 for, respectively. (b) The tabular representation of a number of genes overlapped in tongue cancer across different studies. (c) Schematic representation of commonly up-regulated genes qRT-PCR validation in a cohort of 35 paired tongue tumor samples. The Red denotes up-regulation, blue as downregulation, black as basal expression and gray color; experiment could not be done or results could not be acquired. The ≥2 mean fold change is for up-regulation, ≤0.5 mean fold change for down-regulation and in between 1.99 and 0.501 mean fold change as a basal level expression compared to the adjacent normal tissue sample. (For interpretation of the references to color in this figure legend, the reader is referred to the web version of this article.)

863 of 1693 non-silent somatic variants across 767 genes were predicted to be deleterious (Supplementary Table S4). Further posterior filtering of these variants was performed by restricting to 33 genes found to be significantly mutated using IntOgen (Q-value ≤ 0.05) as potential driver variants (Supplementary Table S5) [16]. Among the HNSCC hallmark genes reported in COSMIC database, we observed recurrent mutations in *TP53* (44%), *NOTCH1* (20%), *CDKN2A* (12%), *HRAS* (12%), *USP6* (8%); while, mutations in *FANCA*, *HLA-A*, *PIK3CA*, *KMT2D* and *PDE4DIP* were observed as non-recurrent (Fig. 1a). Overall the frequency of mutations observed in the hallmark genes were consistent with COSMIC and TCGA HNSCC data with altered frequency for *TP53* and *NOTCH1*, but consistent with reports from ICGC-India (Gingivo-buccal) [28], tongue subsite from India and Asia [29], [30] (Supplementary Table S6). A known mutational signature feature induced by tobacco C:G > A:T transversion was found to be represented in high fraction (53%) (Supplementary Fig. 2b Fig. 2b), which is much higher than observed in various cancers (15–26%) not associated with tobacco [31] and consistent with reports in HNSCC [6], [28].

### Somatic copy number alterations derived from whole exome sequencing data

We used Control-FREEC [32] and cghMCR package to identify genomic regions harboring statistically significant copy number gains and losses relative to normal tissues. 440 amplified and 2275 deleted regions were identified across 23 TSCC patients (Supplementary Tables S7, S8). 18 genes exhibited copy number greater than three (Fig. 1b). Among most frequently amplified regions include 11q13.3 (*CCND1*, *FGF19*, *ORAOV1*, *FADD*); 8q21.3 (*MMP16*), and genes *BOP1* (8q24.3), *EGFR* (33%); *RICTOR* (33%), *PLEC* (33%), *TERT* (26%), *TNK2* (26%), *PIK3CA* and *SOX2* (22%) *MYC* (14%) and *NOTCH1* (14%). Comparative analysis of amplified and deleted gene with previous HNSCC including tongue cancer studies [28], TCGA-HNSCC [6], Vettore et al. [30] and PanCancer [33] revealed statistically significant overlap in the number of genes (Supplementary Table S9).

Additionally, we validated copy number changes in hallmark genes using qPCR (Supplementary Fig. 3). We observed significant co-amplification of *CCND1*, *FGF19*, *ORAOV1*, *FADD* (P-value < 0.01, Co-occurrence); *PIK3CA* and *SOX2* (P-value < 0.001) in this study and TCGA-tongue tumors, which contains genes implicated in cell cycle, cell death/NF-kB pathway and, consistent with previously described in HPV-negative HNSCC tumors [6], [7] (Supplementary Fig. 4a). Interestingly, *EGFR* amplification was significantly mutually exclusive to *CCND1*, *FGF19*, *ORAOV1* and *FADD* amplification (P-value < 0.01, MutEx) including TCGA-tongue cohort (Supplementary Fig. 4a,c) [6], suggesting unique molecular features associated in our study cohort. These novel genetic associations could serve as pathognomonic alterations in HPV-negative early TSCC tumors.

### Whole transcriptome sequencing data identify upregulation of metastases related pathway in early tongue cancer

We performed whole transcriptome sequencing of five adjacent normal, twelve tumor tissue samples to generate an average of 25 and 34 million paired end reads, respectively, that clustered distinctly as shown in Supplementary Fig. 5a–c. Cufflinks [34], a transcript assembler, was used to perform reference guided assembly of the transcripts with an average expression of 11,824 (SD ± 606) genes per sample ≥ 1 FPKM. Of 17 samples that were whole transcriptome sequenced, one normal showed poor distribution of reads (Supplementary Fig. 5a) and tumor samples were misclassified (Supplementary Fig. 5b), were removed them from differential gene expression analysis resulting in 4 normal samples and 11 tumor tissues as shown in Fig. 2a. To identify the differentially expressed genes (DEGs) in whole transcriptome dataset, we used Cuffmerge and Cuffdiff method [34] and applied P-value ≤ 0.05 (Unpaired student-*t*-test) and log fold change 2 as a cut-off to identify 739 significantly differentially expressed genes (Fig. 2a; Supplementary Table S10).

Further, to determine whether gene expression profile in this study was in agreement with the previous studies, we performed a systematic meta-analysis of 4 GEO (microarray) and TCGA (transcriptome sequencing) datasets comprising of 243 tongue tumors and 79 adjacent normal tissue samples expression profile using BRB array toolkit [22] (Supplementary Table S11) and fold change 1.5 and *P-value* ≤ 0.05 was applied as a cutoff to identify the DEGs in each dataset. We identified an average 1281 (SD ± 719) genes to be significantly differentially expressed, where average 619 (SD ± 364) and 662 (SD ± 447) genes showing up and down regulation, respectively (Supplementary Table S11). In overall, the average number of up-regulated genes were comparable with the study based on our cohort (Supplementary Table S11). To identify the commonly deregulated genes across dataset we performed recurrence based comparative analysis across dataset and observed 1146 genes to be deregulated in two or more number of datasets (Supplementary Tables S12 and S13). Among the 1146 deregulated genes, 493 and 653 were showing common upregulation and down-regulation in ≥2 datasets (including this study) in the meta-analysis (Supplementary Fig. 6a). Interestingly, we observed significant overlap i.e. 39% (196/493) up-regulated genes (*P-value* < 0.0001); and 20% (133/653) down regulated genes overlap (*P-value* < 0.0001) with recurrently up-regulated genes in previous datasets (Fig. 2b). Next, to gain broader insight into biological processes related to the commonly DEGs in tongue cancer, we performed gene set enrichment analysis against KEGG gene sets using MSigDB [23]. For the up-regulated genes, significantly enriched KEGG gene sets include several pathways involved in tumor cells metastasis process consistent with previous reports in HNSCC and tongue cancer [35] (Supplementary Fig. 6b, left panel). The down regulated genes, significantly enriched KEGG gene sets include pathways implicated in detoxification of carcinogenic compounds and environmental toxins such as drug metabolism consistent with previous reports in HNSCC, including tongue tumors [35] (Supplementary Fig. 6b, right panel). Interestingly, Arachidonic acid metabolism pathway was previously shown to be down regulated and inactivated via somatic mutations in Indian Gingivobuccal cancer patients [36], suggesting its possible tumor suppressive role via downregulation in tongue cancer patients in this study.

### Upregulation of *MMP10* and other MMPs in early stage tongue primary tumors

**Fig. 3:**
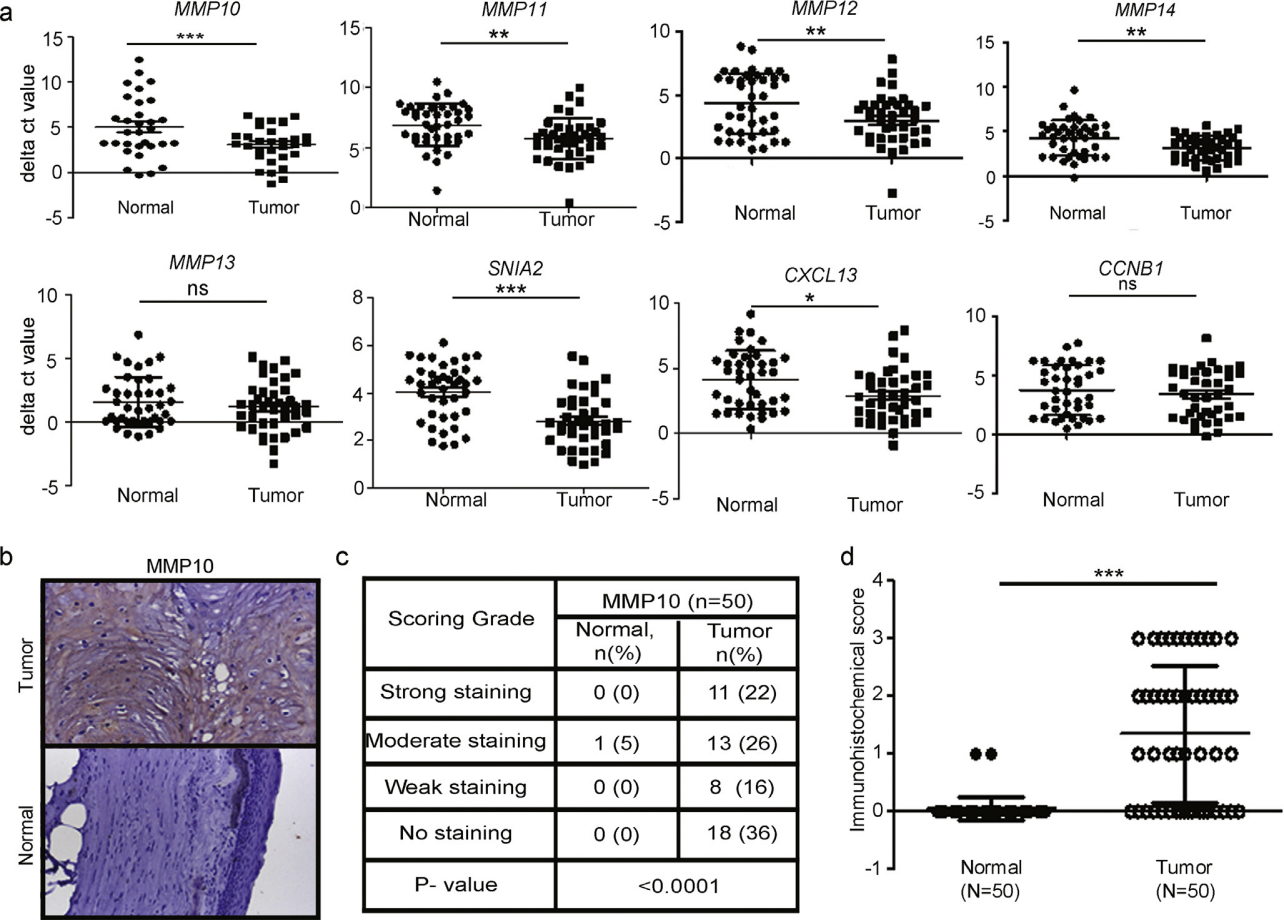
qRT-PCR validation of MMPs and immunohistochemical analysis of MMP10 in early stage tongue cancer. (a) qRT-PCR analysis of *MMP11*, *MMP12*, *MMP13*, *MMP14*, *CXCL13*, *CCNB1*, and *SNAI2* transcript expression in paired normal early tongue tumors (n = 35). Dot plot representation of ΔCt value distribution and its significance between normal and tumors tongue tissue samples for *MMP10*. Each dot represents the average normalized ΔCt value of a gene in a single sample. Median with interquartile range is shown for each gene for normal and tumor samples. P-value is denoted as ^*^; P < 0.01, ^**^; P < 0.001, ^***^; P < 0.0001. (b) Representative IHC stained photomicrographs of tongue tumors and normal samples. The brown color indicates positive expression of MMP10 protein. (c) The tabular representation of different immunohistochemical scores grades of MMP10 in early tongue tumors. P-value is denoted as ^***^; P < 0.0001. The P-value was calculated by Mann-Whitney *U test* using GraphPad Prism 5 program and P-value ≤0.05 was considered as a threshold for statistical significance. (d) Dot plot representation of immunohistochemical score of MMP10 expression in tongue tumors and adjacent normal tissues (n = 50). Each dot represents that final IHC score for each sample and median with interquartile range is shown. Median with interquartile range is shown for MMP10 protein expression in normal and tumor samples. P-value is denoted as ^***^; P < 0.0001.

Several matrix metalloproteinase (MMPs) family genes were among the highly up-regulated genes across ≥3 dataset (Supplementary Table S15). While the role of *MMP1*, *3*, *7* and *9* have previously been described in head and neck cancer [37], [38], [39], we set to ask if remaining 5 of 9 matrix metalloproteinases (*MMP10*, *11*, *12*, *13* and 1*4*) along with 3 hallmark genes *CXCL13*, *CCNB1* and *SNAI2*, as described in Table S12— also play a role in head and neck cancer. The real-time PCR based validations were performed across an additional set of 35 primary paired normal tongue tumors samples. The validated genes were ranked based on their differential expression (Fig. 2c and Fig. 3a). *MMP10* differential expression was most significant (p < 0.0001) that was further validated histochemically (Fig. 3b–d). Incidentally, *MMP10* is known to be involved in promoting metastases [38] and inflammation [40] in other cancers.

Next, we performed immunohistochemical based validation of MMP10 expression in 50 primary early stage paired normal tongue tumors. In the adjacent normal samples, positive MMP10 staining was not observed, whereas, positive cytoplasmic staining of MMP10 was detected in 32/50 (64%) of tongue cancer patients tumors (Fig. 3b), consistent with the previous report in HNSCC tumors [35]. About 48% of primary tongue tumors displayed strong or moderate immunostaining of MMP10 protein, whereas 62% tongue tumors showed weak or no staining (Fig. 3c). In overall, statistically significant differences in immunohistochemical scores were observed in tumors as compared to adjacent normal tissues (P-value < 0.0001, unpaired student-*t*-test) (Fig. 3d) and MMP10 protein was up-regulated in a large proportion of primary tongue tumors.

### Clinical correlation with genetic alterations in early tongue cancer

**Fig. 4:**
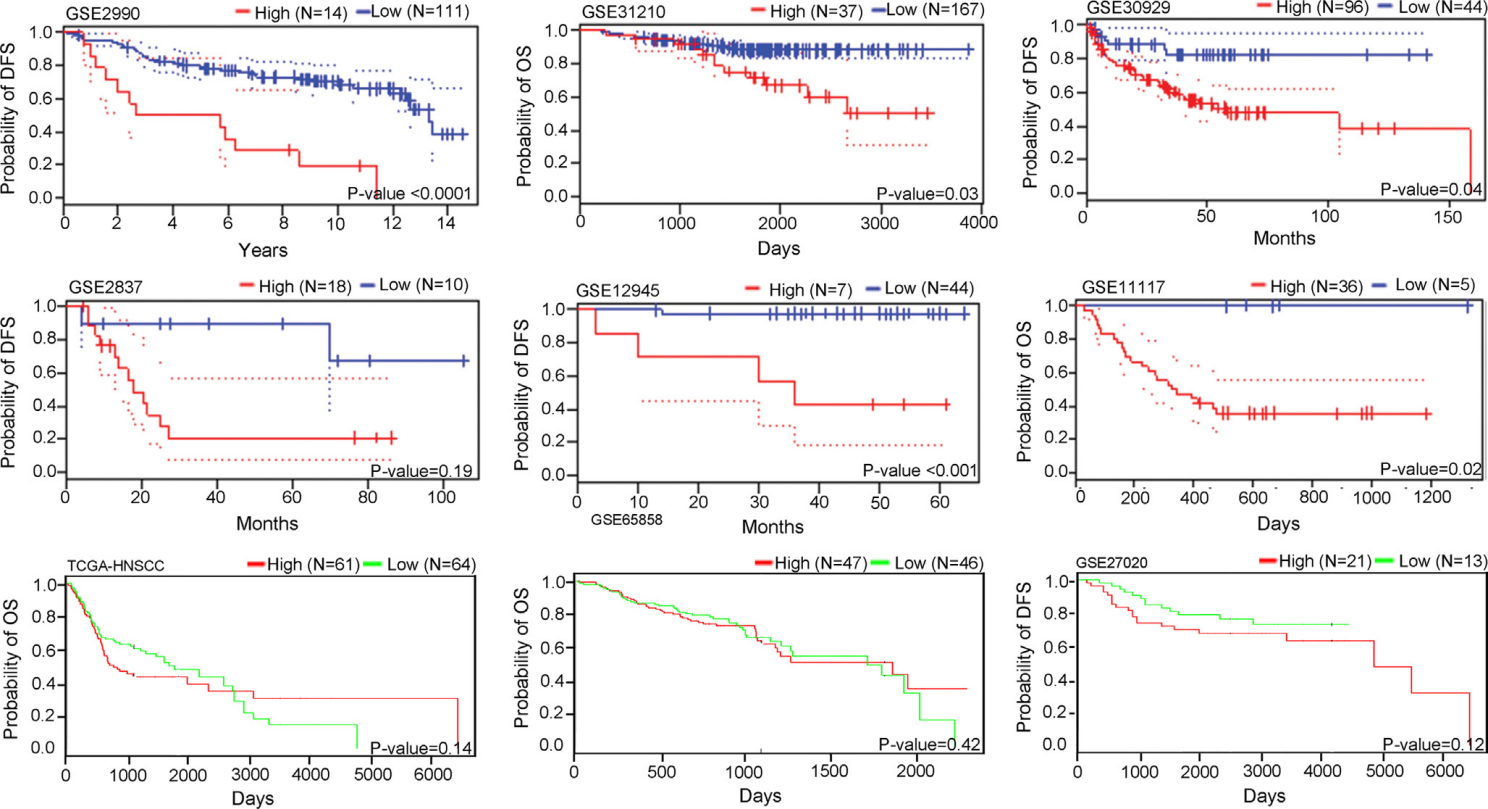
Kaplan-Meier survival curve disease-free survival and overall survival in various cancer types based on the level of *MMP10* gene expression. Kaplan-Meier plot of DFS and OS analysis in HNSCC studies based on low and high *MMP10* gene expression. The red and green lines denote high and low *MMP10* expressing patient’s survival, respectively. The number of samples and median survival in each group is denoted. The log-rank test was applied to accesses the statistical differences in median survival and P-value ≤ 0.05 was considered as statistical significance. (For interpretation of the references to color in this figure legend, the reader is referred to the web version of this article.)

The cohort did not reveal any significant association between clinical features such as age, gender, tumor stage, American Joint committee on Cancer (AJCC) TNM stage, nodal status, smoking, alcohol, tobacco usages with mutations in HNSCC hallmark gene; *TP53*, *NOTCH1*, *CDKN2A*, *CASP8*, *HRAS* and *PIK3CA* (Supplementary Table S14). However, we observed 3 of 3 patients with *HRAS* mutation were tobacco chewers, which is consistent with previous reports in Indian oral cancer patients [41]. As shown in Table 1, an association of *MMP10* transcript and protein expression analyzed in 35 and 43 primary TSCC patients showed a marginal significance with tobacco habit (*P-value* = 0.057). Survival data of these patients were far from maturity. Thus, analysis of GEO gene expression dataset of HNSCC (GSE2837) based on *MMP10* transcript expression performed showed poor survival in the cancer patients with high *MMP10* expression, similar to as observed in breast cancer (GSE2990), lung cancer (GSE31210; 11117), liposarcomas (GSE30929), and colorectal cancer (GSE12945) using PrognoScan [42] and PROGgene [43] (Fig. 4). The association of *MMP10* expression with poor survival in early TSCC remains to be verified in a larger sample set.

**Table 1:** Association of clinical features of early stage TSCC patients with MMP10 transcript and protein level expression.

Clinicopathological features	MMP10 expression, number (%), along the column								
	Variable	N	*MMP10* transcript expression (n = 35)		P value*	N	MMP10 protein expression (n = 43)		P value*
			Basal or Down	Up-regulated			Negative	Positive	
Age	>45 years	15	9 (41%)	6 (46%)	1	21	12 (50%)	9 (47%)	1
	<45 years	20	13 (59%)	7 (54%)		22	12 (50%)	10 (53%)	

Gender	Male	25	17 (77%)	8 (62%)	0.444	30	18 (75%)	12 (63%)	0.509
	Female	10	5 (23%)	5 (38%)		13	6 (25%)	7 (37%)	

AJCC TNM stage	I–II	13	7 (32%)	6 (46%)	0.48	16	8 (33%)	8 (42%)	0.752
	III–IVA	22	15 (68%)	7 (54%)		27	16 (67%)	11 (58%)	
	Node negative	16	7 (54%)	6 (46%)		16	8 (34%)	8 (42%)	

Smoking	Smoker	12	9 (41%)	3 (23%)	0.463	16	8 (33%)	8 (42%)	0.752
	Non-smoker	23	13 (59%)	10 (77%)		27	16 (67%)	11 (58%)	

Alcohol	Yes	8	5 (23%)	3 (23%)	1	14	8 (33%)	6 (32%)	1
	No	27	17 (77%)	10 (77%)		29	16 (67%)	13 (68%)	

Tobacco	Yes	24	18 (82%)	6 (46%)	0.057	28	14 (58%)	14 (74%)	0.349
	No	11	4 (18%)	7 (54%)		15	10 (42%)	5 (27%)	

N: Number of patients.

* Chi-square *t*-test.

## Discussion

Here, we describe the landscape of genomic alterations in a unique set of early staged HPV-negative tobacco or nut chewing tongue cancer patients, using whole exome sequencing and transcriptome sequencing. While lack of survival data is a major limitation of the study, several lines of distinct features underlie this study attributing to unique etiology, subsite, and specific population, which has been previously described for HNSCC [7].

Firstly, the mutational profile of large fraction of patients display high frequency (53%) of C:G > A:T transversion in exome sequencing data—a hallmark of smokeless tobacco usage—reflecting tobacco as the most predominant etiological agent; which is considerably higher than observed (15–26%) in various non-tobacco associated cancer types [31]. We also observed an enriched fraction of C > T transition and C > G transversion, consistent with previous report in gingiva-buccal (ICGC-India) and tongue tumors with tobacco chewing habit [28], [29]. The C > G transversions are known to be caused by tobacco due to reactive oxygen species (8-oxoguanine lesions) and or APOBEC family of cytidine deaminases genes overactivity induced by deamination of 5-methyl-cytosine to uracil in CpG island as described previously. Moreover, the predominance of G > A transition in *TP53* gene in this study (57%; 4/7) is consistent with previous reports in betel quid and tobacco chewing associated oral squamous cell carcinoma tumors from Indian population [44], [45].

Secondly, recent reports suggest the presence of low frequency (∼5%) of RAS mutations in tongue tumors [29], [30]. However, we observed 12% *HRAS* mutations, which though were all tobacco chewers, consistent with previous reports from the Indian population [41]. Similarly, unlike previously reported, inactivating and low-frequency mutation in *NOTCH1* in HNSCC [6], [28], [46], [47], most of the mutations were missense, consistent with a recent report in the Asian population and our report [14]. However, consistent with previous reports, frequent copy number alterations including gains at 5p, 8q, 20q, 22q and 11q and losses at 1p, 5p, 6q, 7p and 21q [6], [28], [29], [30] were significantly represented. Moreover, deleterious somatic variants in HNSCC hallmark genes: *TP53*, *NOTCH1*, *CDKN2A*, *CASP8*, *PIK3CA*, *USP6*, *MLL2*, *HLA-A*, *FANCA*, *PDE4DIP*, and *FAT1* were also identified [29], [30]. Furthermore, significantly co-occurring alterations in *FADD CCND1*, *FGF19*, and *ORAOV1* (P < 0.0001) were found to occur mutually exclusive with *EGFR* amplification among HPV-negative early TSCC tumors, as previously described in other cancers [6], [7]. Interestingly, *EGFR* and *CCND1* oncogenic events are known to act via a common RAS-MAPK Kinase pathway to promote cell cycle and known to act as a driver of oral cancer tumorigenesis [48], [49], [50]. The mutual exclusivity of *EGFR* and *CCND1* amplification suggests activation of a common downstream signalling pathway in different TSCC patients via diverse genetic alterations. Thus this finding may affect the benefit of common downstream inhibitor, such as MAPK inhibitor, to a broader spectrum of patients.

Thirdly, differential gene expression analysis showed significant up-regulation of gene-sets primarily involved in epithelial to mesenchymal transition (EMT) processes, corroborating with known occult lymph node metastasis and invasive behaviour of early stage tongue tumors [51]. Furthermore, meta-analysis approaches of gene expression studies lead to a precise estimation of recurrently expressed genes across data set. The overexpression of MMPs has been known to be involved in ECM degradation thereby facilitating the process of tumor invasion and metastasis leading to an aggressive course of disease in HNSCC patients [37]. qRT-PCR validation across 35 paired normal early stage primary tumors for up-regulated genes (*MMP10*, *MMP11*, *MMP12*, *MMP14*, *CXCL13*, *CCNB1*, *SNIA2*) showed significant up-regulation in tumors suggesting reliability of genes identified from this study. Of the multiple MMPs found to be up-regulated, immunohistochemical analysis of MMP10 in 50 paired normal early stage primary tumors showed significant up-regulation of protein expression in primary tumors owing its possible role in early stage progression as described in other cancer types as a potential prognostic biomarker to stratify those likely to develop metastases [52], [53]. However, insights about their specific role await validation in a larger independent cohort with survival followed by functional analysis.
