# Supplementary material for: Genomic characterization of tobacco/nut chewing HPV-negative early stage tongue tumors identify MMP10 as a candidate to predict metastases
Source: Oral Oncol. 2017 Oct;73:56–64. doi: 10.1016/j.oraloncology.2017.08.003 (PMC5628952; doi:10.1016/j.oraloncology.2017.08.003)
Supplement: Supplementary data 1 [file mmc1.docx]

**Supplementary Figures legends**

**Supplementary Fig. S1**

**Schematic representation of study overview.**

**(a)** Integrated analysis involves somatic point mutations and DNA copy number changes and gene expression changes identified from transcriptome sequencing (**b)** Sample distribution in different study such as exome, transcriptome sequencing, and validation of copy number changes and differential expression. The black and white filled boxes denotes patient sample included and excluded for respective analysis, respectively. In the exome sequencing track, asterisk * denotes tumors sample which were not included for variant analysis due to low coverage and/or poor correlation with their matched normal based on SNP profiler analysis. The asterisk ** denotes unpaired tumor sample.

**Supplementary Fig. S2**

**Various characteristic features of somatic variants identified from whole exome sequencing data.**

**(a)** Pie-chart representation percent frequency of dbSNP, TMC-SNPdb, COSMICdb, and novel of variants identified in exome sequencing of early tongue tumors. (**b)** Distribution of number and frequency of various substitutions in exome sequencing variants. Bar graph representation of percentage frequency of transition and transversions in early tongue tumors. (**c)** Doughnut plots representation of somatic coding and non-coding variants. (**d)** Variant classification of somatic coding and non-coding variants. Bar plot representation of coding variants (left panel) and non-coding variants (right panel) percentage frequency distribution in various types of categories.

**Supplementary Fig. S3**

**DNA copy number validation using qPCR in early tongue tumor samples.**

The DNA copy number alterations were validated using qPCR. The solid red filled; amplification, blue; deletion and black; diploid and gray, could not be determined. Percentage frequencies for each gene are denoted.

**Supplementary Fig. S4**

**Genetic association analysis of hallmark genes copy number alterations and somatic variants in tongue tumors.**

**(a & b)** Schematic representation of copy number alterations and mutation in HNSCC hallmark genes in this study (n=23). **(c)** Schematic representation of copy number alterations and mutation in HNSCC hallmark genes in TCGA-tongue tumor cohort (n=79). The red filled box denotes amplification, blue; deletion and black; mutations.

**Supplementary Fig. S5**

**Quality control analysis of mRNA sequencing data.**

**(a)** Box pot representation of log10 (FPKM) values for each samples to accesses the overall distribution. **(b)** Dendrogram representation using unsupervised hierarchical clustering of each samples based on global gene expression profile. Normal and tumor samples are distinctly clustered besides two samples (Sample N5 and T8) showing mixed behaviour. **(c)** Density plot showing the bell shaped curve suggesting the uniform distribution of transcriptome sequencing across samples for each gene. Sample N5 and T8 were found to be outlier and showing unusual pattern, hence not included in differential expression analysis, but included in other analysis.

**Supplementary Fig. S6**

**Commonly deregulated gene and pathways in tongue cancer.**

**(a)** Venn diagram to illustrate the overlapped of significantly differentially expressed and commonly up (left panel) and down (right panel) regulated genes in at least two gene expression studies. **(b)** Bar plot representation of significantly deregulated gene sets in tongue cancer identified from meta-analysis.
