# Supplementary material for: Genomic characterization of tobacco/nut chewing HPV-negative early stage tongue tumors identify MMP10 as a candidate to predict metastases
Source: Oral Oncol. 2017 Oct;73:56–64. doi: 10.1016/j.oraloncology.2017.08.003 (PMC5628952; doi:10.1016/j.oraloncology.2017.08.003)
Supplement: Supplementary data 2 [file mmc2.docx]

**Supplementary Material and Methods**

**Identification of somatic variants**

In brief, paired-end Raw sequence reads generated were mapped to the human reference genome (build hg19) using BWA v. 0.6.2 [1] and PCR duplicates were removed using Picard tools v.1.74 (http:broadinstitute.github.io/picard/). Base quality score recalibration and indel realignment were performed and variants were called from each sample separately using GATK (version 2.5-2) [2, 3] Unified Genotyper and MuTect (v1.0.27783) [4]. The variants called from GATK for each patient tumor were further filtered against its matched normal sample. The variants obtained from MuTect and GATK were later merged and variants having ≥5 reads supporting altered base were considered for further analysis. To reduce the possibility of misclassification of germline variations as somatic mutation all the variants obtained from tumor were further filtered against Indian specific germline database (TMC-SNPdb) [5] to remove ethnic specific germline variants. Later, all the mutation were annotated using Oncotator (v1.1.6.0). Using in-house developed script, we compared and identified variants present in dbSNP (v142)[6] and COSMIC database (v68)[7]. Post filtration of variations present in the dbSNP database, only somatic COSMIC mutation, and novel mutations were obtained. The functional prediction tool based analysis for non-synonymous variants was performed using nine different such as tools: dbNSFP v2.0 (includes SIFT, Polyphen2_HDIV, Polyphen2_HVAR, LRT, Mutation Taster, Mutation Accessor and FATHMM)[8], CanDRA (v1.0)[9], and PROVEAN[10]. Mutations which were predicted to be potentially deleterious by ≥4 prediction algorithm were further used for analysis. We also filtered mutation in those genes which are not expressed in our TSCC transcriptome data. Comparative analysis of genes harboring potentially deleterious mutations was performed with COSMIC Cancer gene census database[7], PanCancer gene list (significantly mutated genes across 12 cancer type)[11], and TCGA HNSCC mutation dataset using cBioPortal [12]. Total number of identified somatic substitutions in exome sequencing were extracted from MutSigCV output and were processed to calculate the number and frequency distribution of various transitions and transversions.

**Somatic copy number analysis from Exome sequencing data**

In brief, normal and tumor BAM files were processed to get raw read counts for each target region defined in the bed file using Control-FREEC [13]. Conversion of counts to copy number ratio was done and segmentation was performed using lasso method. Annotation and post-processing were carried out using R programming and we also combined the segmentation results for 23 early TSCC patient samples. For identification of genes with statistically significant copy number changes, we used cghMCR (<http://www.bioconductor.org/packages/2.3/bioc/html/cghMCR.html>), an R implementation of a modified version of GISTIC analysis [14]. The cghMCR package usages a Segments-of-Gain-Or-Loss (SGOL) score to specify the frequency and degree of copy number changes in each gene or segment. Three standard deviations (SDs) from the mean SGOL score was selected because 97.17% of genes had SGOL scores within this range, hence, a gene with SGOL score ≥4 defined as amplified genes and ≤-2 as deleted genes.

**Transcriptome sequencing and data analysis to identify expressed genes**

Briefly, mRNA was purified from 4µg of intact total RNA using oligodT beads (TruSeq RNA Sample Preparation Kit, Illumina). Qualitative and quantitative analysis of cDNA libraries were performed using High Sensitivity DNA chip on 2100 Bioanalyzer (Agilent) and qPCR with KAPA Library Quant Kit (Kapa Biosystems) in ABI 7900HT system (Life Technologies), respectively. cDNA libraries were loaded on Illumina flow cell (version 3) to generate clusters using TruSeq PE (Paired-End) Cluster Kit v3-cBot-HS kit and clustered flow was sequenced for 201 cycles on HiSeq-1500 System (Illumina) using TruSeq PE Cluster Kit v3 and TruSeq SBS Kit v3 (Illumina) to generate at least 30 million reads per sample. Post sequencing, De-multiplexing was carried out on raw sequencing data was performed on basis of index sequences using CASAVA (version 1.8.4, Illumina).

# **Immunohistochemical analysis**

In brief, paraffin section slides were placed in microwave for by incubation at 58^o^C for 30 minutes, followed by deparaffinization, rehydration, and quenching steps. The antigen retrieval was performed in a pressure cooker (1 whistle) with citrate buffer at pH 6.0 followed by cooling at room temperature (RT). 1:50 diluted horse serum from Vectashield Kit (Vector Laboratories, USA) was used for the blocking. The specificity of antibody was determined using western blot analysis. Additionally, secondary control and positive control as mouse kidney tissue was used to optimize the dilution for MMP10 antibody. Tissue sections were incubated overnight at 4^o^C with the anti-MMP10 (1:100) primary antibody (Abcam; ab59437). Next day, slides were rinsed with wash buffer and incubated for the 30 minutes with biotinylated secondary antibody provided with Vectashield kit, followed by tertiary antibody (Reagent A & B) incubation for 1 hour. The chromogenic reaction was carried out with 3,3′- diaminobenzidine chromogen solution for 5 minutes resulting in brown color signal as expected. Later, slides were rinsed with deionized water and counterstained with hematoxylin, dehydrated and mounted with the DPX mounting reagent and cover slip.

References:

[1] Li H, Durbin R. Fast and accurate short read alignment with Burrows-Wheeler transform. Bioinformatics. 2009;25:1754-60.

[2] McKenna A, Hanna M, Banks E, Sivachenko A, Cibulskis K, Kernytsky A, et al. The Genome Analysis Toolkit: a MapReduce framework for analyzing next-generation DNA sequencing data. Genome Res. 2010;20:1297-303.

[3] DePristo MA, Banks E, Poplin R, Garimella KV, Maguire JR, Hartl C, et al. A framework for variation discovery and genotyping using next-generation DNA sequencing data. Nat Genet. 2011;43:491-8.

[4] Cibulskis K, Lawrence MS, Carter SL, Sivachenko A, Jaffe D, Sougnez C, et al. Sensitive detection of somatic point mutations in impure and heterogeneous cancer samples. Nat Biotechnol. 2013;31:213-9.

[5] Upadhyay P GN, Desai S*, Sahoo B, Singh A,Togar T, Iyer P, Prasad R, Chandrani P, Gupta S, Dutt A. TMC-SNPdb: an Indian germline variant database derived from whole exome sequences. Database (Oxford). 2016.

[6] Sherry ST, Ward MH, Kholodov M, Baker J, Phan L, Smigielski EM, et al. dbSNP: the NCBI database of genetic variation. Nucleic acids research. 2001;29:308-11.

[7] Forbes SA, Bhamra G, Bamford S, Dawson E, Kok C, Clements J, et al. The Catalogue of Somatic Mutations in Cancer (COSMIC). Current protocols in human genetics / editorial board, Jonathan L Haines [et al]. 2008;Chapter 10:Unit 10 1.

[8] Liu X, Jian X, Boerwinkle E. dbNSFP v2.0: a database of human non-synonymous SNVs and their functional predictions and annotations. Hum Mutat. 2013;34:E2393-402.

[9] Mao Y, Chen H, Liang H, Meric-Bernstam F, Mills GB, Chen K. CanDrA: cancer-specific driver missense mutation annotation with optimized features. PLoS ONE. 2013;8:e77945.

[10] Choi Y, Chan AP. PROVEAN web server: a tool to predict the functional effect of amino acid substitutions and indels. Bioinformatics. 2015;31:2745-7.

[11] Kandoth C, McLellan MD, Vandin F, Ye K, Niu B, Lu C, et al. Mutational landscape and significance across 12 major cancer types. Nature. 2013;502:333-9.

[12] Cancer Genome Atlas N. Comprehensive genomic characterization of head and neck squamous cell carcinomas. Nature. 2015;517:576-82.

[13] Boeva V, Popova T, Bleakley K, Chiche P, Cappo J, Schleiermacher G, et al. Control-FREEC: a tool for assessing copy number and allelic content using next-generation sequencing data. Bioinformatics. 2012;28:423-5.

[14] Beroukhim R, Getz G, Nghiemphu L, Barretina J, Hsueh T, Linhart D, et al. Assessing the significance of chromosomal aberrations in cancer: methodology and application to glioma. Proceedings of the National Academy of Sciences of the United States of America. 2007;104:20007-12.
