# Supplementary material for: Genomic characterization of tobacco/nut chewing HPV-negative early stage tongue tumors identify MMP10 as a candidate to predict metastases
Source: Oral Oncol. 2017 Oct;73:56–64. doi: 10.1016/j.oraloncology.2017.08.003 (PMC5628952; doi:10.1016/j.oraloncology.2017.08.003)

Supplementary Figure S1

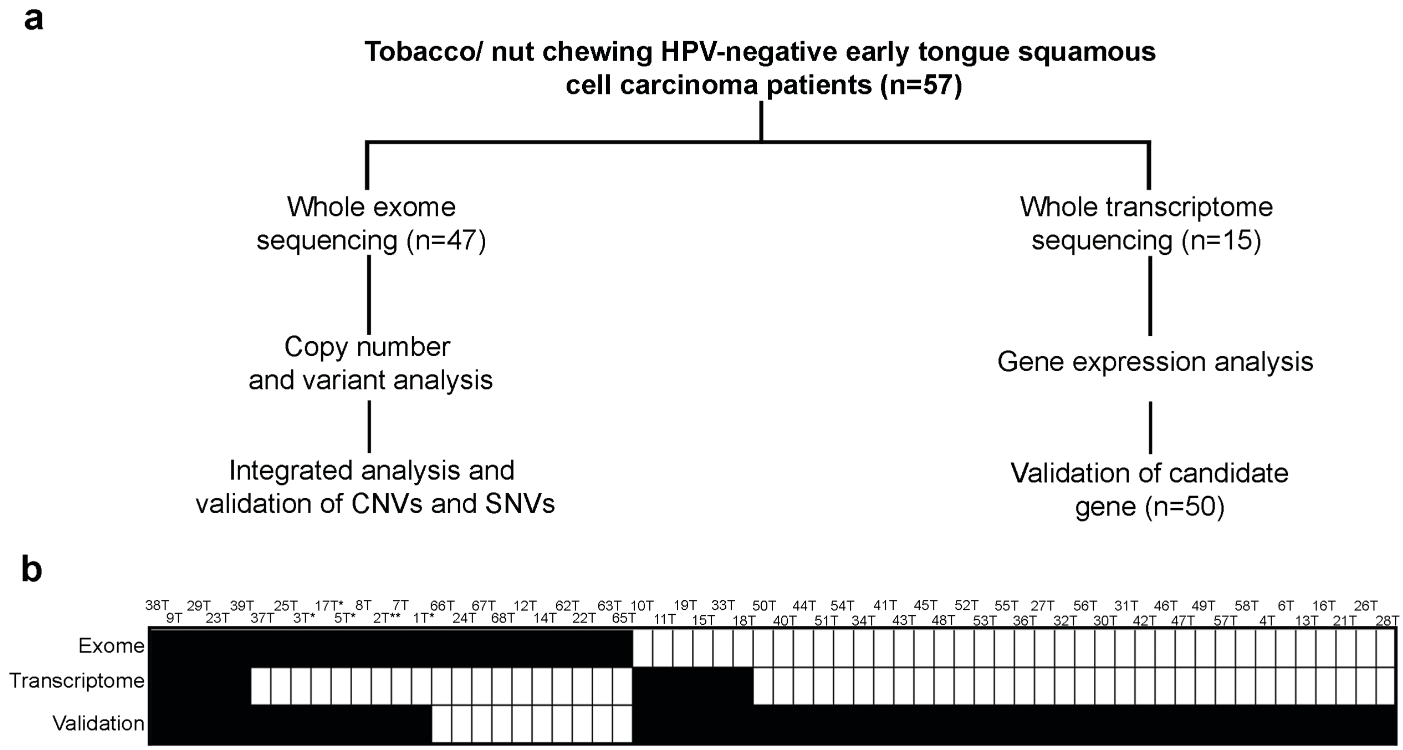

Supplementary Figure S2

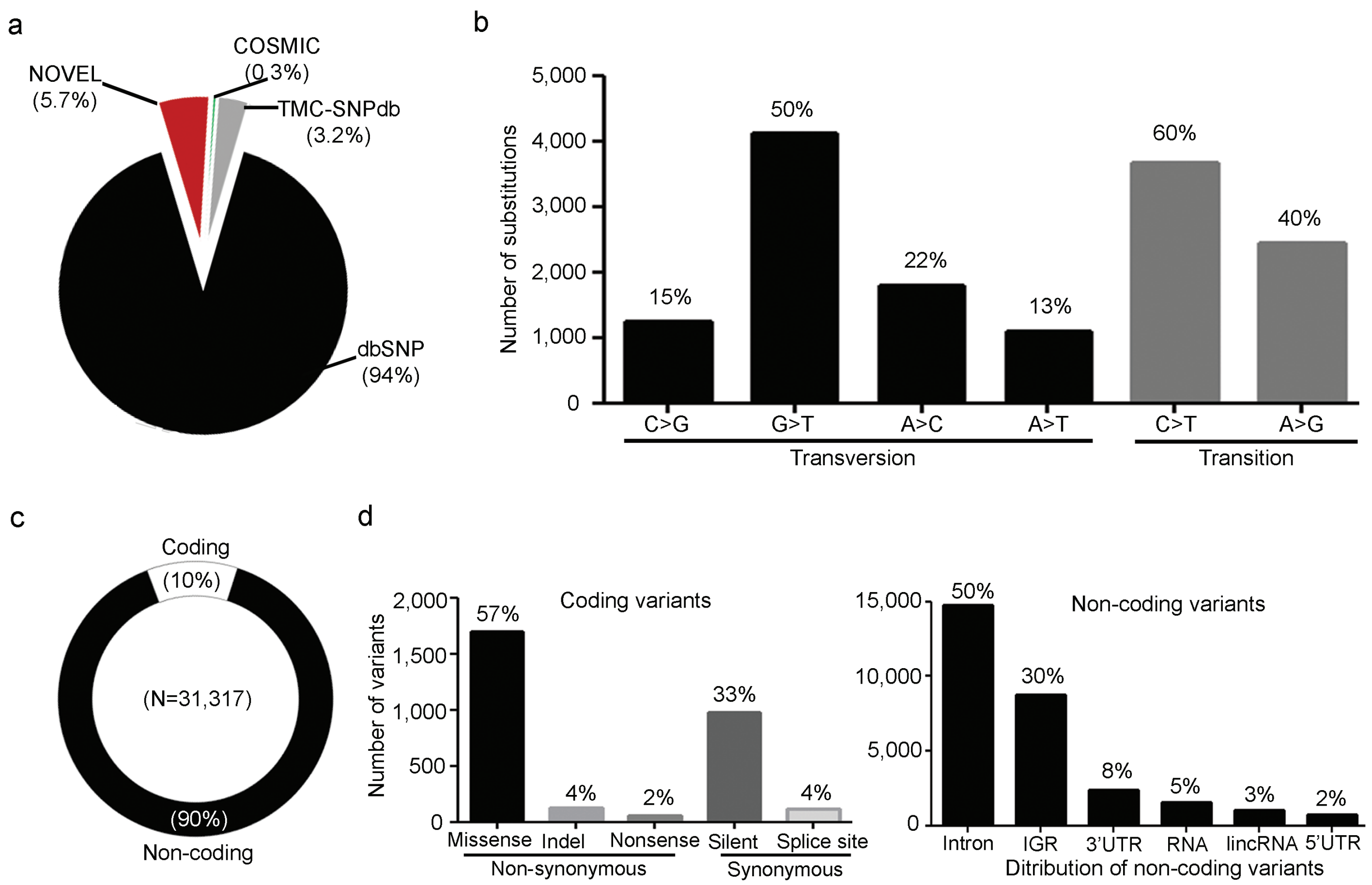

Supplementary Figure S3

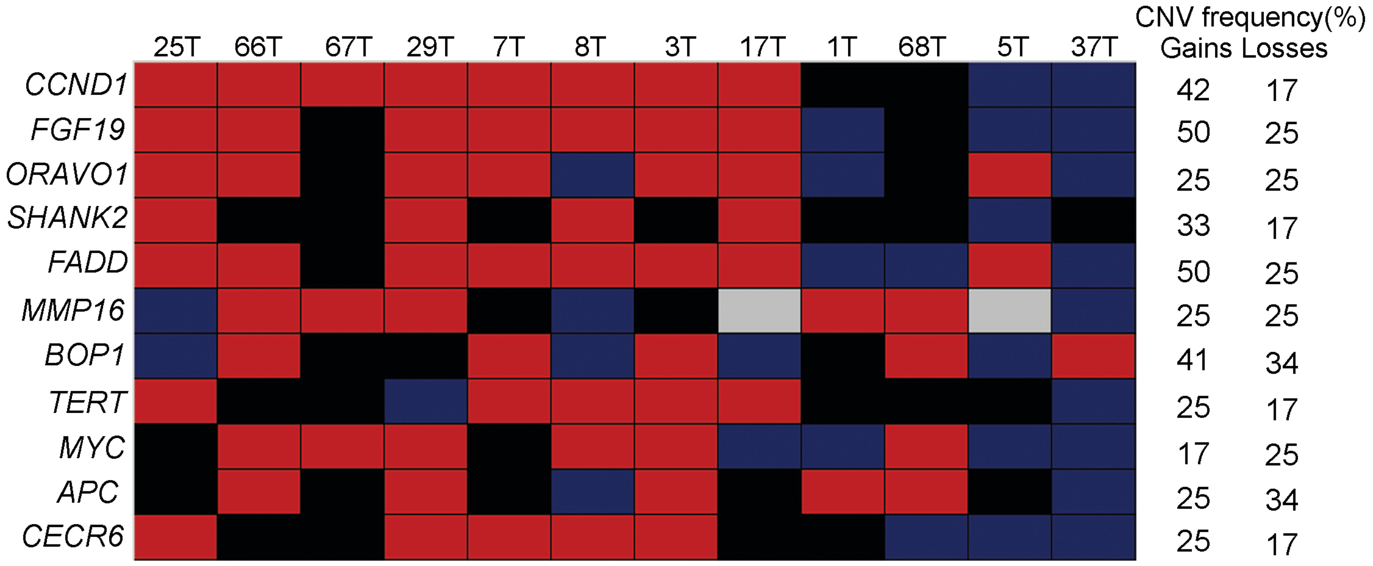

Supplementary Figure S4

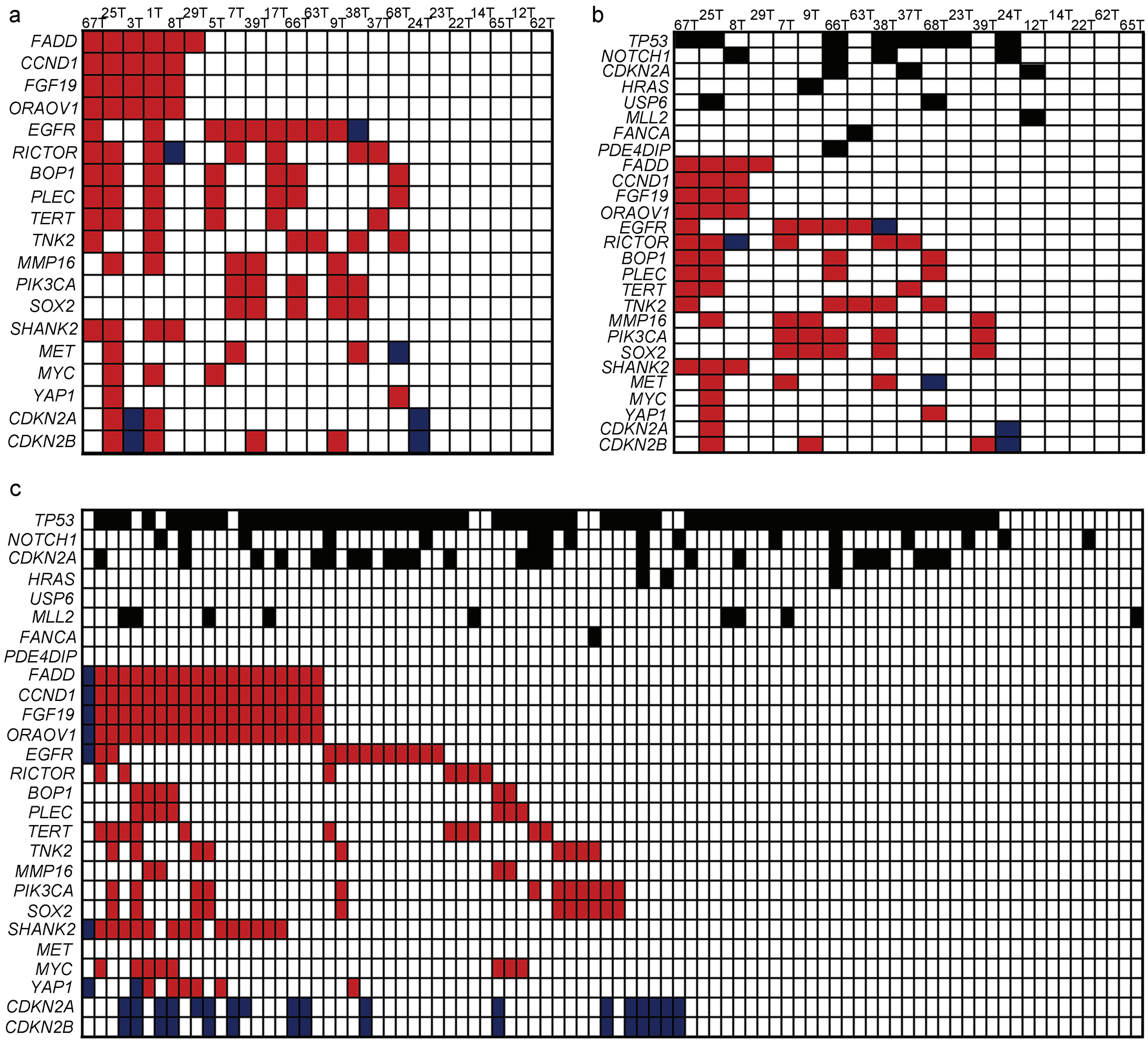

Supplementary Figure S5

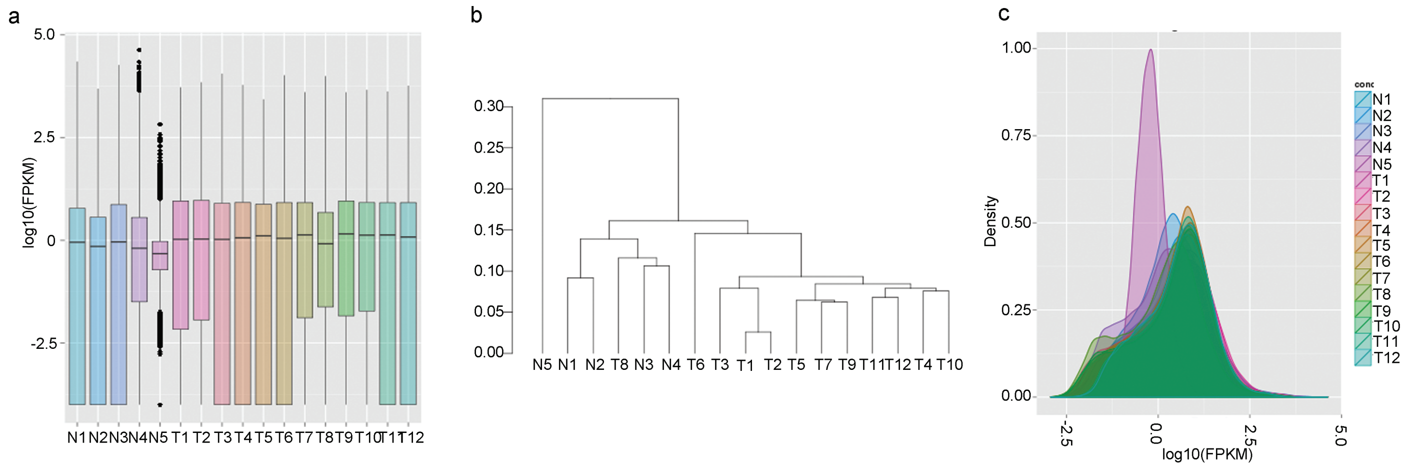

Supplementary Figure S6

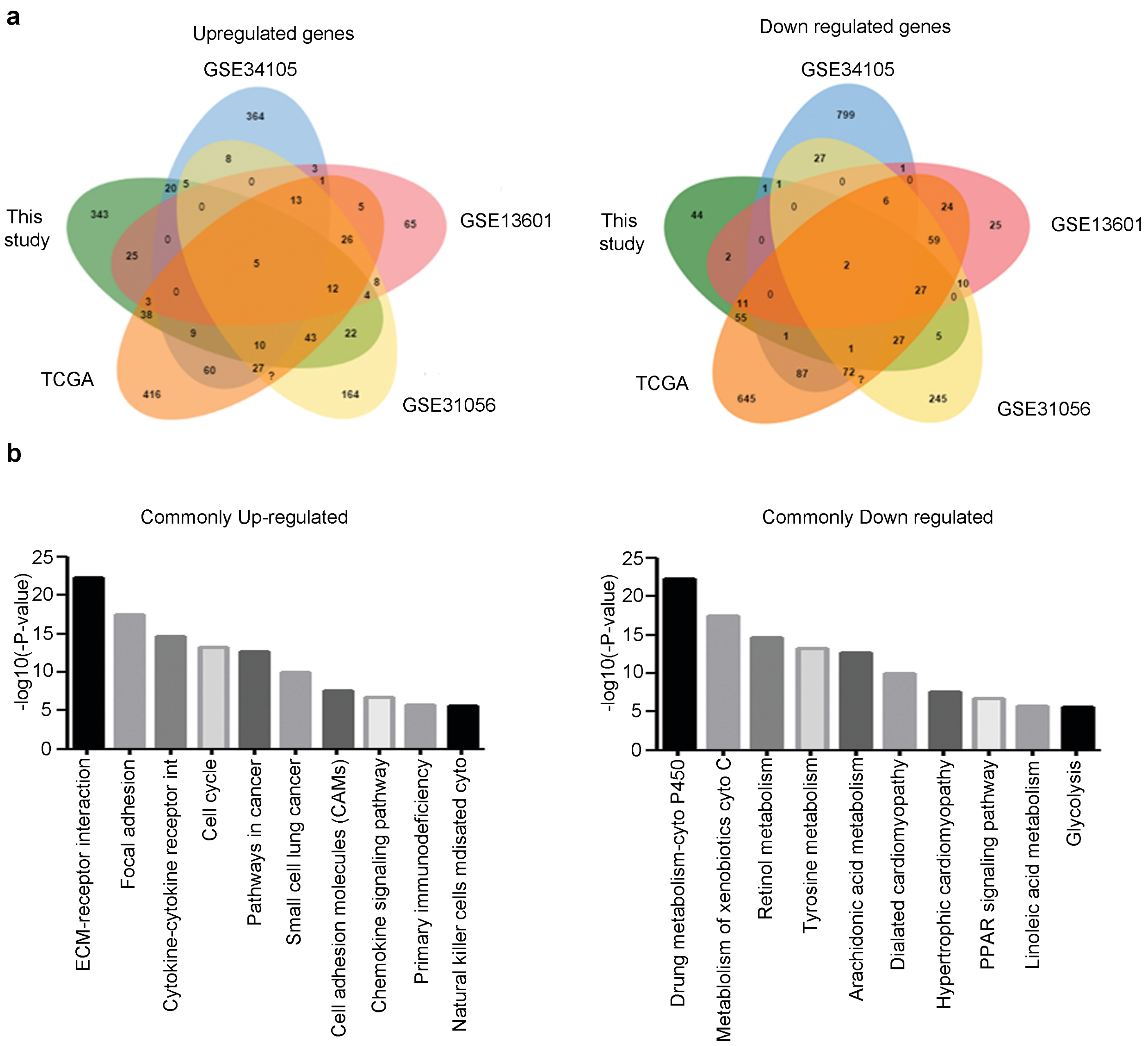

Supplement: Supplementary data 4 [file mmc4.pdf]
